# Nurse Implementation of Music for Pain Relief in the Inpatient Setting

**DOI:** 10.1155/nrp/7578595

**Published:** 2026-08-29

**Authors:** Heather Watson, Alexandra Johnson, Yea-Jen Hsu, Zehui Zhou, Elizabeth Scala, Kerry Devlin, Lauren E. Russell, Kyurim Kang, Vinciya Pandian, Alexander Pantelyat, Jane Sumergrade Webster, Jill A. Marsteller

**Affiliations:** ^1^ Department of Nursing, Center for Nursing Inquiry, Johns Hopkins Hospital, 600 N. Wolfe Street, Baltimore 21287, Maryland, USA, jhu.edu; ^2^ Johns Hopkins Armstrong Institute for Patient Safety and Quality, 750 E Pratt Street, Baltimore 21202, Maryland, USA; ^3^ Department of Health Policy and Management, Johns Hopkins University Bloomberg School of Public Health, 615 N Wolfe Street, Baltimore 21205, Maryland, USA, jhu.edu; ^4^ Brown University, 1 Prospect Street, Providence 02912, Rhode Island, USA, brown.edu; ^5^ Center for Music and Medicine, Johns Hopkins University School of Medicine, Carnegie Building, 601 N Wolfe St #518-D, Baltimore 21287, Maryland, USA, jhu.edu; ^6^ Office of Health Equity, Veterans Health Administration, U.S. Department of Veterans Affairs, 810 Vermont Avenue Northwest, Washington 20420, District of Columbia, USA; ^7^ The Pennsylvania State University, Penn State Ross and Carole Nese College of Nursing, 201 Nursing Sciences Building, University Park 16802, Pennsylvania, USA, psu.edu; ^8^ Johns Hopkins Hospital, Baltimore, Maryland, USA, jhu.edu

**Keywords:** adjunct therapy, implementation science, music listening, nurse practice, pain, patient advocacy

## Abstract

**Background:**

The music for pain relief study tested an innovative broad hypothesis that nurses can effectively integrate the use of music as an option for pain and anxiety management among their patients. The literature supports music for pain relief as an established best practice, and implementing music listening at the bedside supports patient‐centered care and autonomous nursing practice.

**Methods:**

Guided by the Practical, Robust Implementation, and Sustainability Model (PRISM), a multidisciplinary research team comprised of nurse researchers, a patient/family co‐investigator, and implementation scientists engaged nurses to implement the protocol on four inpatient units—three adult and one pediatric—from July 2021 to December 2021. Implementation strategies included nurse champions, in‐person training, a unit research binder, and Spotify music streaming accounts with pre‐established playlists. Reach, effectiveness, adoption, implementation, and maintenance (RE‐AIM) using data from the electronic health record (EHR), a bedside form, and a pre–post survey of nurse motivation to implement was assessed.

**Results:**

EHR data showed improvement in the monthly average documented music provision from 9 sessions preintervention to 16 postintervention and 22 in the sustainment phase. Bedside forms (*n* = 32) revealed nurses offered recorded music to alleviate anxiety (32%), boredom (27%), pain (22%), or procedural distraction (19%). Patients averaged 78 min of music listening, most typically on provided tablets (78%). Patients selected the genre in 46% of plays. Nurse perceptions towards the intervention significantly improved with respect to having accomplished something worthwhile. Pain scores captured before and after music listening did not show statistically significant differences.

**Conclusions:**

Nurses successfully integrated the evidence‐based practice of offering recorded music to their patients during routine care, potentially facilitating pain and anxiety relief. The nurse’s attitudes toward offering music showed they felt it was worthwhile. Implementation during the COVID‐19 pandemic may have increased data collection fatigue, which limited postimplementation data collection.

## 1. Background

Nurse management of pain in hospital settings is an important feature of their interactions with patients. Nurses provide comfort and a listening ear as they perform clinical services for patients, including delivering pain medication. Pain management strategies available to nurses, who have arguably the most “touch time” with patients of any of the hospital staff, are nonpharmacological in nature. Pharmacologic interventions—including opioid analgesics—continue to be the mainstay of pain management in acute care settings and can be beneficial when used appropriately [1]. However, overreliance on medications as the sole approach to pain management presents challenges for both clinicians and patients alike. For clinicians, interval dosing based on standing orders may not effectively manage patients’ pain in a timely manner, and nonpharmacological pain management options are limited. For patients, there is a risk of developing dependence on analgesics, namely, opioids and potential long‐term concomitant risks associated with dependence. These factors create a sense of urgency to address how pain is managed in the hospital setting. A promising area of innovation is nurse employment of underused but effective nonpharmacological pain management strategies at the bedside to optimize patient care and potentially decrease the use of pharmacological agents, especially opioids. Music listening is a promising intervention receiving attention in health care today.

Our review of the literature related to music‐based interventions as a nonpharmacological pain management strategy yielded consistent results. According to a recent randomized controlled trial (RCT), three 15‐min music listening sessions resulted in a reduction in postoperative cardiac pain (as measured by the visual analog scale) and anxiety (via state‐trait anxiety inventory) as well as improvement in vital signs when compared to a control group that did not listen to music [2]. Another RCT study in postoperative sternotomy patients determined that listening to patient preferred songs for 15–30 min four times a day for up to seven postoperative days reduced the mean scores of pain (as measured by a numerical pain rating scale) and anxiety (via postoperative anxiety scale) and improved vital signs compared to a control group with noise canceling headphones and no music [3].

Moreover, a recent mixed‐method study found that change scores between baseline and Week 12 showed that group vocal music therapy (VMT) sessions (eight 60‐min sessions) were effective in managing chronic pain in patients with arthritis, degenerative disc/spinal stenosis, neuropathy, and fibromyalgia [4]. The intervention included breathing exercises, body awareness, music making, verbal processing, singing a song, and lyrics discussion [4]. There were medium‐sized effects for pain intensity and small effects for pain interference [4]. The participants reported that VMT supported self‐management of pain, enhanced psychological well‐being, and strengthened relationships with others [4]. Additionally, music‐based interventions were suggested for managing neuropathic pain [4]. Several theoretical approaches which view singing as a multisensory communication tool have suggested that parents sing to infants to manage neonatal pain [5]. Overall, the literature finds that music‐based interventions have a positive impact on reducing pain scores.

The music for pain relief (M4PR) study sought to expand nursing capabilities to help patients cope with pain and advance the implementation science literature by testing approaches to effectively incorporate offering recorded music into nursing workflow. We tested the broad hypothesis that nurses can implement recorded music as an option for pain management among their patients to redirect attention and reduce patients’ perception of pain. Furthermore, we explored when and how music was offered to patients and assessed nurse attitudes toward offering music.

## 2. Methods

The study team conducted this implementation science pilot study in two phases. In phase one, after having successfully added music to the nurses’ pain intervention pull‐down menu in the electronic health record (EHR), we directly observed the existing practice of music delivery to patients in January of 2020, yielding 22 observations, and summarized EHR evidence of the use of music. We also collected perceived barriers and facilitators to the use of music listening as an intervention for pain management through stakeholder engagement meetings (unpublished data, 2020).

In the second phase, reported here, we developed an implementation protocol informed by the observations and stakeholder feedback, established unit‐based teams with nurse champions, and piloted implementation of the M4PR protocol in four inpatient units in a large, urban academic hospital. The study received approval from the Institutional Review Board (IRB00232556).

### 2.1. Conceptual Framework

**FIGURE 1:**
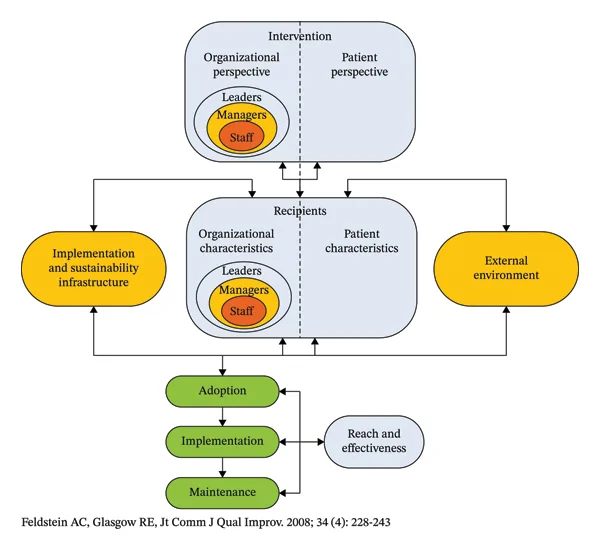
The practical, robust implementation and sustainability model (PRISM).

The study used the Practical, Robust Implementation, and Sustainability Model (PRISM), a comprehensive model of the implementation process, to guide the pilot study and our evaluation (Figure 1) [6, 7]. PRISM [7] combines key concepts from existing quality improvement and implementation science frameworks and models, including Rogers’ diffusion of innovations theory [8], the PRECEDE/PROCEED model [9], Chronic Care Model [10], Promoting Action on Research Implementation in Health Services (PARiHS) [11], the Model for Improvement [12], Six Sigma [13], and the RE‐AIM framework [14]. The result is a comprehensive model positing that the adoption, implementation, and maintenance of an intervention are affected by perceived usability and acceptability of the intervention, characteristics of the recipients, the external environment, and existing sustainability and implementation infrastructure.

Our approach included studying several of the RE‐AIM outcomes of the PRISM framework including reach, effectiveness, adoption, implementation (fidelity), and maintenance (Table 1) [7, 14].

**TABLE 1:** RE‐AIM outcomes as measured in M4PR.

Implementation outcome	Measured as
Reach	Count of music offerings among those eligible
Effectiveness	Comparison of average pain scores pre‐ and post‐music by location of pain, among those receiving music
Adoption	Training participation; motivation to implement
Implementation	Count of study contacts with frontline staff; music modality selected; why music was offered
Maintenance	Count of music offerings among those eligible during sustainment period

### 2.2. Setting

We recruited staff from four inpatient units—three adult and one pediatric—in a large, urban academic hospital. None of the units were designated as COVID‐19 units, although the M4PR implementation occurred during the Delta‐variant wave in 2021. Unit selection was voluntary. Members of the research team attended a meeting of hospital‐based nurse managers (pre‐COVID) to present information on the research study. Managers responded with interest, and the study team invited them to participate in the M4PR pilot.

### 2.3. Identifying Implementation Barriers and Selecting Facilitation Strategies

The study team identified barriers and facilitators through observation and individual meetings with the pilot unit nurse managers and classified them according to the Theoretical Domains Framework (TDF) [15, 16]. The team then mapped TDF [16] categories to the implementation strategies using the “Select Tool” (see Table 2) [17]. The team used these results to develop the plan for intervention training and implementation. The major barriers to using recorded music identified by nurse managers were lack of available resources (e.g., tablets and playlists), beliefs about capabilities of nurses to implement, and beliefs about impact on patients’ perception of pain. The major strategies we employed to address these barriers were education through informative meetings; training, using direct contact and train‐the‐trainer approaches; an example workflow; persuasion and modeling using unit champions; and resources through donated Spotify accounts.

**TABLE 2:** Select tool: matched implementation strategies to address identified barriers to implementation.

TDF domain	Intervention function	Implementation strategies	Definitions
Knowledge Social Professional Role Beliefs about Capabilities Goals Beliefs about Consequences	Education	Conduct educational meetings Distribute educational materials	Hold meetings involving program targets (e.g., providers, administrators, other organizational stakeholders, and community, patient/consumer, and family stakeholders) to improve knowledge about the ideal practice Distribute educational materials (e.g., guidelines, manuals, and toolkits) in person, by mail, and/or electronically to improve knowledge about the ideal practice

Cognitive and interpersonal skills Beliefs about capabilities Goals Environmental context and resources	Enablement	Use of champions Prepare patients/consumers to be active participants	Identify and prepare individuals to dedicate themselves to supporting, marketing, and overcoming indifference or resistance related to implementing the ideal practice Prepare patients/consumers to be active in their care, e.g., to ask questions about the ideal practice and evidence behind the ideal practice

Cognitive and interpersonal skills Social Professional Role Beliefs about capabilities Goals Beliefs about Consequences	Modeling	Model and simulate change Shadow other experts	Have experts/leaders/respected colleagues’ model or simulate the ideal practice Provide ways for designated individuals from the target stakeholders group(s) to directly observe other experienced people perform the ideal practice

Social Professional Role Beliefs about capabilities Goals Beliefs about Consequences	Persuasion	Identify and use local opinion leaders Use mass media	Inform providers identified by colleagues as opinion leaders or “educationally influential” about the ideal practice in the hopes that they will influence colleagues to adopt it Use media to reach large numbers of people to spread the word about the ideal practice

Cognitive and interpersonal skills Identity Environmental context and resources	Training	Use train‐the‐trainer strategies Conduct training	Train designated providers or organizations so that they can train others in the ideal practice Train providers on how to perform the ideal practice in a “hands‐on” manner

### 2.4. Participants

All nurses and other staff who could offer music‐based interventions for pain management or other purposes (e.g., boredom and anxiety) were included. All patients admitted to the four units in the study eligible for music listening were included and excluded patients were those with conditions that prevent the use of music (e.g., nonhearing and stimuli sensitivity).

### 2.5. Music Curation

A music therapist on the research team created Spotify playlists that reflected 8 different musical genres to maximize opportunities for choice‐making when patients were available to self‐select music during the implementation phase (see Supporting Appendix A). While there is no universal set of musical features or style of music that *guarantees* decreased arousal for all music listeners [18], playlists were designed to reflect music selection criteria widely reported in recorded music listening interventions for pain management and relaxation induction [19]. Selection criteria included (1) selection of musical pieces without song lyrics [20] and without abrupt rhythmic or dynamic changes to prevent sudden arousal or startle response [21]; (2) selection of songs with tempi of approximately 60–80 beats‐per‐minute, which aligns with the lower end of the range for average resting heart rate in most healthy adults [22]; and (3) a gradual decrease in song tempi across the duration of each playlist to promote rhythmic entrainment, which may lead to decreased physiologic arousal [23, 24].

The study team included a member of the institution’s Pediatric Patient and Family Advisory Council (PPFAC), who was instrumental in acquiring the donated Spotify accounts used during implementation. Each unit was given enough Spotify accounts to access the predetermined playlists without overlapping with other patients. Patients were encouraged to select from the predetermined recorded music playlists via in‐room tablets but could opt for their own playlists if desired. Televisions in the patient rooms also could play audio‐only and provided a listening option for patients not interested in using the tablets to listen to music. There were no standardized playlists accessible via television.

### 2.6. Implementation Strategies

Prior to implementation, study team members engaged with nurse managers and staff from May 2021 through June 2021, introducing the study and answering initial questions. Nurses and other staff participated in education for the protocol’s implementation. Education occurred through multiple modalities. Members of the research team attended staff meetings and unit huddles to present the study and provided a link to online educational materials to engage unit staff. Prior to education and champion training, the study team obtained oral consent and provided a quick response (QR) code link to the attitudinal survey and the bedside data collection form.

Each unit had a nurse champion who encouraged their peers to offer recorded music and fill out the bedside form. Unit champions received additional training with details on the evidence supporting the intervention and hands‐on and how‐to demonstrations of the use of tablets or patient’s personal devices to access preset playlists using Spotify. They were a resource for the nurses who had questions about the workflow, and they role modeled offering recorded M4PR with their patients. Unit champions participated in ongoing unit huddles and staff meeting to promote the study.

**FIGURE 2:**
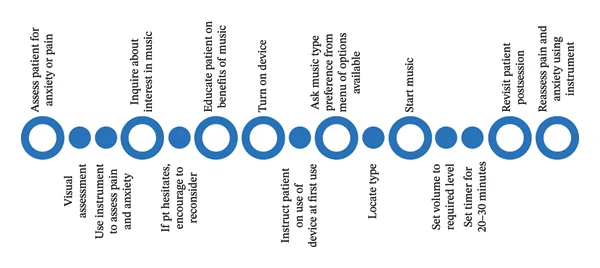
Process flow.

The study team provided a sample workflow to help nurses offer recorded music in a study binder on each unit (see Figure 2). From July 2021 to December 2021, nurses approached their patients as per usual care to assess the patient’s pain and anxiety levels. Regardless of the pain/anxiety score, the nurse or support staff offered recorded music while noting this intervention would not affect the timing of the patient’s pain medication regimen. Patients were encouraged to listen to music for at least 30 min but were not restricted if they wanted a shorter or longer period. If patients had their own headphones, they were able to use them as needed and the study team did not provide headphones to the patients due to infection and cost concerns. The patient’s pain and anxiety levels were to be reassessed within 1 hour of the music listening intervention. Clinicians placed a door sign created by the study team on the patient’s door requesting visitors and other clinicians not to enter the room during the music listening intervention duration. Additionally, music listening was not limited to patients experiencing pain or anxiety but was offered to patients at the nurses’ discretion (e.g., as a remedy for boredom or restlessness or in response to specific requests to listen to music), and there were no designated procedures associated with the intervention. On some units, other staff, including technicians and customer service representatives, were engaged to assist with setting up the music delivery on available devices (e.g., iPads and TV).

In addition to the sample workflow, a study team member performed regular check‐ins on the pilot units to provide ongoing support to the staff.

### 2.7. Data Collection and Analysis

Data collection for this pilot study included (1) survey data from primarily frontline nurses regarding their perceptions of the recorded music intervention using a Qualtrics online electronic survey with embedded consent; (2) a brief bedside data sheet filled by nurses (Bedside Report Form); and (3) EHR data for documentation of music offered and pain/anxiety scores using a waiver of consent.

#### 2.7.1. Attitudinal Surveys

We assessed nurses’ motivation to implement the project using a validated questionnaire based on Vroom’s model of motivation [25]. This model conceptualizes motivation as a combination of valence, instrumentality, and expectancy. Expectancy (E) was determined by asking respondents to rate their belief in the likelihood of the project’s successful implementation when effort is applied in their practice. Instrumentality (I) was evaluated by assessing respondents′ perceptions of how the project, upon successful implementation, would contribute to achieving seven potential goals. Valence (V) was assessed by asking respondents to rate the importance of each of these seven goals. We included in the survey motivation to implement regarding specific potential outcomes of the intervention (e.g., involving patients in their own care, patient satisfaction with care, costs of care, and reductions in opioid use).

We first described the characteristics of respondents who completed the survey at baseline and during the postintervention period using frequencies and percentages. The attitudinal survey consisted of 15‐, 5‐, or 7‐point Likert scaled questions, ranging from “strongly disagree” to “strongly agree” or “not important” to “extremely important.” Motivation was calculated as a multiplicative score of VxIxE. To evaluate changes in their motivation, ordinal logistic regression models were conducted, adjusting for respondents’ age, gender, race, unit, and role. A clustered sandwich estimator was utilized to account for repeated measures from the same individuals. We hypothesize that motivation to implement will average below midpoint in the scale at baseline but will change positively by the end of the intervention period.

#### 2.7.2. Bedside Report Form

The form collects information on the type of recorded music selected, reason for use of recorded music, and anxiety score (if not recorded elsewhere). Research staff data‐entered quantitative items in the Bedside Report Form and summarized the data using percentages and means.

#### 2.7.3. EHR Data

To assess the impact of the music listening intervention on patients′ pain, we extracted and compared pain scores within 1 h before the documented music intervention and within 3 h after for patients who received the intervention. We employed Wilcoxon signed‐rank tests for the paired data to compare pain scores overall and by their pain locations. All analyses were conducted using Stata version 18.0 [26].

### 2.8. Results

#### 2.8.1. EHR Data Overall Characteristics

There were 4073 hospital admissions identified in the participating units during the study period. Approximately 10% of the encounters were for patients under 18 years old and 23% were for patients aged 65 and older. The majority of the patients were male (53%), White (54%), and non‐Hispanic (93%). We did not find these demographic characteristics to be statistically different across the three intervention phases (see Table 3).

**TABLE 3:** Characteristics of patients in the participating units by intervention phase.

	All		Preintervention		Postintervention		Sustainment period		*p* value
	*n* = 4073	%	*n* = 1001	%	*n* = 1906	%	*n* = 1166	%	
Age									
5–17	392	9.6	74	7.4	196	10.3	122	10.5	0.072
18–24	232	5.7	54	5.4	123	6.5	55	4.7	
25–34	382	9.4	106	10.6	163	8.6	113	9.7	
35–44	412	10.1	109	10.9	185	9.7	118	10.1	
45–54	523	12.8	142	14.2	227	11.9	154	13.2	
55–64	792	19.5	189	18.9	371	19.5	232	19.9	
65–74	815	20.0	202	20.2	402	21.1	211	18.1	
75+	525	12.9	125	12.5	239	12.5	161	13.8	
Gender									
Female	1900	46.7	462	46.2	881	46.2	557	47.8	0.899
Male	2170	53.3	538	53.8	1024	53.7	608	52.1	
Unknown	3	0.1	1	0.1	1	0.1	1	0.1	
Race									
White or Caucasian	2180	53.5	547	54.7	1021	53.6	612	52.5	
Black or African American	1434	35.2	360	36.0	665	34.9	409	35.1	
Asian	158	3.9	28	2.8	75	3.9	55	4.7	0.148
Other	288	7.1	60	6.0	142	7.5	86	7.4	
Unknown	13	0.3	6	0.6	3	0.2	4	0.3	
Ethnicity									
Hispanic or Latino	254	6.2	54	5.4	123	6.5	77	6.6	0.200
Not Hispanic or Latino	3801	93.3	943	94.2	1778	93.3	1080	92.6	
Unknown	18	0.4	4	0.4	5	0.3	9	0.8	

#### 2.8.2. Survey Respondent Characteristics

One hundred and six nurses responded to the baseline survey (response rate = 56%) and 41 responded to the postintervention survey (response rate = 21%). Of the 147 total respondents to the attitudinal survey (see Table 4), the majority were White, female, with an average of five years of work experience. The group included primarily nurses, with a mean age of 31 years. Among respondents, 43.5% worked in the pediatric inpatient unit and 56.5% in the adult inpatient units.

**TABLE 4:** Characteristics of attitudinal survey respondents.

	Preintervention		Postintervention		*p* value^1^
	*n* = 106	%	*n* = 41	%	
Unit					
Bloomberg 9N	46	43.4	18	43.9	0.208
Meyer 3	18	17.0	4	9.8	
Zayed 11W	21	19.8	5	12.2	
Zayed 12W	21	19.8	14	34.2	
Role					
Nurse/nurse manager	90	84.9	37	90.2	0.179
Technician	10	9.4	2	4.9	
CCSC/CCSR	5	4.7	0	0.0	
Other	1	0.9	2	4.9	
Experience in current position					
Less than 1 year	14	13.2	9	22.0	0.526
2‐3 years	35	33.0	10	24.4	
3–9 years	38	35.9	14	34.2	
10+ years	19	17.9	8	19.5	
Age					
20–44 years	85	80.2	35	85.4	0.878
45+ years	14	13.2	4	9.8	
Decline to answer	7	6.6	2	4.9	
Gender					
Female	93	87.7	34	82.9	0.350
Male	10	9.4	7	17.1	
Decline to answer	3	2.8	0	0.0	
Race					
White	69	65.1	25	61.0	0.430
Black	19	17.9	8	19.5	
Other	13	12.3	8	19.5	
Decline to answer	5	4.7	0	0.0	

^1^From chi‐square tests or Fisher’s exact tests.

#### 2.8.3. Reach

**FIGURE 3:**
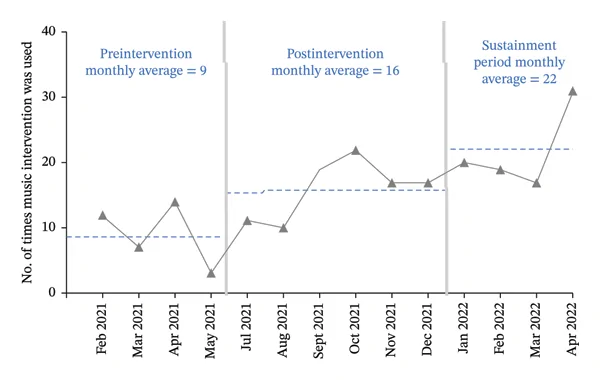
Documented monthly use of music interventions by intervention phase.

We treated all patients seen in the four participating units as eligible to receive recorded M4PR, boredom, or anxiety. Thus, among those eligible, EHR data showed a total of 26 patients received music intervention before implementation compared to 103 patients during implementation, demonstrating a 296% increase. The monthly averages increased from 9 sessions preintervention to 16 postintervention (see Figure 3; adult/child‐stratified results are displayed in Supporting Figure 1).

#### 2.8.4. Effectiveness

We did not observe statistically significant changes in pain scores before and after the music listening intervention, neither overall nor when analyzed by specific pain locations (see Table 5 for the overall results and Supporting Table 1 for results stratified by adults and children).

**TABLE 5:** Comparison of pain scores before and after receiving music interventions.

	n	Within 1 h before music intervention		Within 3 h after music intervention		*p* value^1^
		Mean	SD	Mean	SD	
All	43	5.14	2.99	5.33	3.50	0.868
Abdomen	7	6.57	1.99	7.43	2.94	0.610
Extremities	6	3.83	1.83	4.67	2.94	0.831
Generalized	4	8.00	2.83	7.50	3.79	0.317
HEENT	7	4.79	1.63	5.57	3.69	0.553
Thorax	6	5.50	2.59	6.17	3.19	0.898
Unknown	13	4.12	3.96	3.31	3.35	0.246

^1^From Wilcoxon signed‐rank tests.

#### 2.8.5. Adoption

The four pilot units had a total staff census of 187 registered nurses, and 127 received education; there were an additional 20 support staff (e.g., technicians and registrars) who received education. The total staff trained is based upon noted attendance; however, it is important to consider that staff were able to view video presentations on their own time and could receive further educational opportunities through champions, so it is possible more staff participated in education.

Table 6 illustrates that nurse perceptions and attitudes toward offering recorded music, as measured by motivation to implement M4PR, showed significant change (*p* ≤ 0.05) from pre‐ to postimplementation in one item, helping the nurses feel that they were doing something worthwhile. Two additional measures showed improvement trending toward significance (*p* < 0.1) involving patients in their care and feeling of autonomy in practice. All means increased except the importance of reducing opioid dependency among the patient population.

**TABLE 6:** Pre–post comparison of provider and staff reported motivation scores^1^.

	Preintervention			Postintervention			Ordinal logistic regression^2^	
	n	Mean	SD	n	Mean	SD	OR	95% CI
Reducing patients’ use of pain medication	101	136.9	53.4	37	145.8	47.2	1.38	(0.70 − 2.73)
Improving patient/client satisfaction with care	101	157.9	53.7	37	162.6	47.1	1.24	(0.64 − 2.41)
Involving patients in their own care	103	154.7	54.8	37	167.6	49.0	1.91	(0.98 − 3.71)^∗^
Reducing cost of patient care	98	132.3	58.7	37	139.1	49.3	1.50	(0.80 − 2.84)
Expanding autonomy of practice	99	136.9	57.7	36	148.1	49.2	1.90	(0.98 − 3.67)^∗^
Contributing to the reduction of opioid dependency among our patient population	100	140.5	56.5	38	136.7	41.3	0.83	(0.45 − 1.52)
Feeling of having accomplished something worthwhile	101	146.3	52.5	36	163.6	46.6	2.19	(1.12 − 4.30)^∗∗^

^1^Generated by multiplying expectancy (coded as 1–7), instrumentality (coded as 1–7), and valence (coded as 1–5).

^2^Adjusted for age, gender, race, unit, and role (repeated measure was accounted for using clustered sandwich estimator).

^∗^< 0.1.

^∗∗^< 0.05.

#### 2.8.6. Implementation

Between May 2021 and December 2021, the study team logged contact with the pilot units over 40 times through emails, Zoom calls, and in‐person visits. The use of champions and nurse manager support also facilitated the implementation of music at the bedside.

Bedside Report Forms (*n* = 32) revealed that nurses offered recorded music for anxiety relief (31.7%), boredom (26.8%), pain relief (22%), and for procedural distraction (19.5%). Patients listened to music for an average of 78 min on hospital‐provided tablets (78%), personal devices (15%), or television (7%). The genre was selected by patients (46%), staff (43%), and visitors (11%) (data not shown.)

#### 2.8.7. Maintenance

The Spotify accounts provided to the units were time limited, but free accounts could still be accessed through hospital‐provided tablets or through patients’ personal devices until the end of the sustainment period (May 2022). During the sustainment period, recorded music was offered on average 22 times per month. Documentation of recorded music offered remained steady throughout the sustainment period, but there was a sharp increase in documentation of recorded music listening use after March 2022 (Figure 3).

### 2.9. Discussion

Music listening has been shown as an effective adjunct therapy for pain management [27, 28]. We sought to determine the implementation outcomes when nurses sought to include offering recorded music in their daily patient workflow and assessed how nurse motivation to implement changed over time. We found that even during crises, like a pandemic, nurses were able to integrate recorded music into patient care although documented use was somewhat infrequent compared to the number of eligible patients. Nurses did not consistently document nonpharmacological interventions within the patient’s EHR, however. Documentation burden is a chronic problem within healthcare [28], so it is common to find that certain interventions are not accounted for in the EHR, especially if they are not tied to a regulatory requirement. Creating an environment where nurses can easily direct patients to recorded music listening as an option, without subsequently adding greater burden on workflow, could increase use of music listening. Accessing tablets for use was inconsistent across units and the design of the tablet cases made hearing the recorded music more challenging. Stressors related to staffing during the pandemic may have decreased the ability for nurses to engage in the study procedures [29].

Establishing the “why” of an intervention is critical to facilitating successful implementation [30, 31]; as nurses indicated at baseline, one of the most selected reasons to perform the intervention was to improve patient care. Nurses also indicated the importance of autonomous practice opportunities, favoring the ability to provide patient relief options within their scope and not relying on another provider to prescribe the intervention. Although not significantly, nurses’ attitudes about the intervention improved due to the positive feedback they received from their patients. Focusing on aspects of nurse autonomy and positive patient perception of the intervention may be the key to encouraging broader adoption and implementation.

Patients who participated in listening to music frequently used the hospital‐provided tablet, indicating that having the tablet available helped facilitate access to the music intervention. Additionally, patients most often chose their own genre of music versus the predetermined playlists and listened longer than the minimum amount of 30 min. Providing choice for the patients encouraged participation and may foster a sense of control in an otherwise powerless environment. Including a member of the pediatric PFAC provided invaluable insights of the patient perspective and facilitated the ability to provide music through Spotify accounts. Easy access to music could impact the sustainability of the intervention. The donated accounts were time limited; organizations may consider music subscription services at the institutional level to provide resources, facilitate ease of use, and promote maintenance. Anecdotally, champions suggested music was offered far more often, but documentation burden was a barrier to collecting the data.

The implementation occurred during the COVID‐19 pandemic, which included a period of limited patient visitation. It was noted that there was an increase in the use of music in the sustainability period and that corresponded with the easing of visitor restrictions in the hospital setting as pandemic numbers decreased over time.

Another aspect of the study was to determine whether the use of the M4PR protocol guiding music‐based interventions for pain reduces the perception of pain, reduces anxiety, and decreases the use of pharmacological agents for pain control. The evidence in the literature [27, 32–34] is clear that music has these benefits; however, the results from this intervention did not demonstrate statistically significant reductions in pain or anxiety as documented in the EMR. As previously noted, this was likely due to inconsistent documentation practices that limited the ability to connect the music intervention with the pain scores. The Bedside Report Forms collected indicated that music was used for anxiety relief more often than pain relief. However, there was not a standard measure to capture anxiety in the EHR.

#### 2.9.1. Limitations

There are several limitations to acknowledge in this study. Since the study was a small pilot in one institution, the results are not broadly generalizable. However, the research team sought to increase the ability to translate findings by recruiting four test units that varied in the types of inpatients served, including psychiatric and medical units, adult, and pediatric. Another limitation was that we were unable to track music offered independently from documentation in the EHR. Although we requested nurses fill out a Bedside Report Form, use of this tracking tool was limited, and we did not have a one‐to‐one match of forms to EHR‐based documentation of music delivery. Anecdotal information from nurse managers and champions suggested that music was used more often than documented. Further, the interruption of the pilot due to the COVID‐19 pandemic and staffing challenges severely limited capacity to adequately capture postimplementation survey data. Unfortunately, the Bedside Report Forms were not consistently completed for all the patients who received music as an intervention, limiting the results. In testing whether pain was lessened by listening to music in our study, we were unable to link specific pairs of pain scores from EHR to instances of music offer. This structural limitation in the EHR limited our ability to assess music effectiveness.

## 3. Conclusions

The use of M4PR is a well‐established best practice for reducing pain and anxiety in a clinical setting [2, 3, 32–34]. This pilot study of bedside nurses′ implementation of M4PR in an inpatient setting for adult and pediatric populations demonstrated some success and opportunities for future research. Although there are multiple barriers to overcome, there were also facilitators that showed promise for future implementation efforts. This low‐cost nonpharmacologic adjunctive modality is an attractive option for hospitals that are continually burdened by budget constraints and regulatory measures. Nurses have an ardent desire to promote patient care and to provide adjunct treatments within their scope of practice. The music listening intervention helps support nurses in their efforts to promote patient comfort. Additionally, creating environments where patients and families are given a measure control over their care and well‐being strongly aligns with patient‐centered care values.

## Author Contributions

H. W.: data curation, investigation, project administration, resources, supervision, validation, visualization, writing–original draft, and writing–review and editing. A. J.: data curation, investigation, project administration, visualization, and writing–review and editing. Y. H.: formal analysis and writing–review and editing. Z. Z.: formal analysis. E. S.: conceptualization and funding acquisition. K. D.: resources and writing–review and editing. L. E. R: writing–review and editing. K. K.: writing–review and editing. V. P.: conceptualization, funding acquisition, and writing–review and editing. A. P.: conceptualization, resources, and writing–review and editing. J. S. W.: conceptualization, resources, and writing–review and editing. J. A. M.: conceptualization, funding acquisition, methodology, resources, supervision, writing–original draft, and writing–review and editing.

## Funding

The funding for the workgroup breakouts came from the Johns Hopkins Discovery Award program.

## Disclosure

There is a statistician on the author team; Yea‐Jen Hsu, PhD MHA. All authors read and approved the final manuscript. The funder had no role in the design, collection, analysis, and interpretation of data; in the writing of the manuscript; or in the decision to submit this manuscript for publication.

## Ethics Statement

This study was conducted in accordance with the Declaration of Helsinki (2013), the Belmont Report, and the U.S. Common Rule (45 CFR 46). The Johns Hopkins Medicaine Institutional Review Board reviewed the protocol (protocol #IRB00232556, approval date 2/28/2020) and determined it to be minimal risk, granting a waiver of informed consent under 45 CFR 46.116 and a waiver of HIPAA authorization as applicable under 45 CFR 164.512(i). Only de‐identified or limited data sets were used as approved. Approval to conduct the study was gained from the Institutional Review Board (IRB00232556).

## Consent

All participants provided informed consent before participating in the study, which included consent to publish anonymous quotes from individual participants.

## Conflicts of Interest

Dr. Vinciya Pandian serves as a member of the Journal of Clinical Nursing Editorial Board. This author had no role in editorial review, selection of reviewers, or decision‐making regarding this manuscript.

The other authors declare no conflicts of interest.

## Supporting Information

Additional supporting information can be found online in the Supporting Information section.

## Supporting information


**Supporting Information 1** Supporting Figure 1. Stratified documented monthly use of music interventions by intervention phase. This figure displays monthly documented use of music interventions among adult and pediatric patients before and after implementation. Use increased during the postintervention phase, with the greatest and most sustained increase observed among adult patients, while pediatric use remained lower and more variable.

**Supporting Information 2** Supporting Table 1. Stratified pain scores before and after music intervention. This table presents pain scores recorded within 1 h before and within 3 h after the music intervention, stratified by age group and pain location. No statistically significant differences were observed in pain scores overall or within any subgroup.

**Supporting Information 3** Supporting Appendix A: Examples of Curated Playlists for the Music for Pain Relief Research Study. This appendix provides examples of the curated Spotify playlists offered during the Music for Pain Relief study. The playlists included relaxation music for children and instrumental selections featuring acoustic guitar, ocean sounds, and cello, with durations ranging from 31 to 35 min.

## Data Availability

The datasets generated and/or analyzed during the current study are not publicly available due to the proprietary nature of patient care data but are available from the corresponding author upon reasonable request.

## References

[1] Chou R. W. J. , Ahmed A. Y. , Blazina I. et al., Treatments for Acute Pain: a Systematic Review. Comparative Effectiveness Review No. 240, 2020, Agency for Healthcare Research and Quality.

[2] Dong Y. , Zhang L. , Chen L. W. , and Luo Z. R. , Music Therapy for Pain and Anxiety in Patients After Cardiac Valve Replacement: A Randomized Controlled Clinical Trial, BMC Cardiovascular Disorders. (2023) 23, no. 1, 10.1186/s12872-023-03058-5. PMC9845817 36650441

[3] Ganesan P. , Manjini K. J. , and Bathala Vedagiri S. C. , Effect of Music on Pain, Anxiety and Physiological Parameters Among Postoperative Sternotomy Patients: A Randomized Controlled Trial, Journal of Caring Sciences. (2022) 11, no. 3, 139–147, 10.34172/jcs.2022.18. 36247036 PMC9526790

[4] Low M. Y. , Lacson C. , Zhang F. , Kesslick A. , and Bradt J. , Vocal Music Therapy for Chronic Pain: A Mixed Methods Feasibility Study, Journal of Alternative & Complementary Medicine. (2020) 26, no. 2, 113–122, 10.1089/acm.2019.0249. 31750726 PMC7044781

[5] Ullsten A. , Eriksson M. , Klässbo M. , and Volgsten U. , Singing, Sharing, Soothing – Biopsychosocial Rationales for Parental infant-directed Singing in Neonatal Pain Management: A Theoretical Approach, Music & Science. (2018) 1, 10.1177/2059204318780841.

[6] McCreight M. S. , Rabin B. A. , Glasgow R. E. et al., Using the Practical, Robust Implementation and Sustainability Model (PRISM) to Qualitatively Assess Multilevel Contextual Factors to Help Plan, Implement, Evaluate, and Disseminate Health Services Programs, Translational Behavioral Medicine. (2019) 9, no. 6, 1002–1011, 10.1093/tbm/ibz085. 31170296

[7] Feldstein A. C. and Glasgow R. E. , A Practical, Robust Implementation and Sustainability Model (PRISM) for Integrating Research Findings into Practice, Joint Commission Journal on Quality and Patient Safety. (2008) 34, no. 4, 228–243, 10.1016/s1553-7250(08)34030-6. 18468362

[8] Rogers E. M. , The Diffusion of Innovations, 2003, 5th edition, The Free Press.

[9] Crosby R. and Noar S. M. , What is a Planning Model? An Introduction to PRECEDE-PROCEED, Journal of Public Health Dentistry. (2011) 71, no. Suppl 1, S7–S15, 10.1111/j.1752-7325.2011.00235.x. 21656942

[10] Wagner E. H. , Bennett S. M. , Austin B. T. , Greene S. M. , Schaefer J. K. , and Vonkorff M. , Finding Common Ground: Patient-Centeredness and Evidence-based Chronic Illness Care, Journal of Alternative & Complementary Medicine. (2005) 11, no. Suppl 1, S7–S15, 10.1089/acm.2005.11.s-7. 16332190

[11] Kitson A. , Harvey G. , and McCormack B. , Enabling the Implementation of Evidence Based Practice: a Conceptual Framework, Quality in Health Care. (1998) 7, no. 3, 149–158, 10.1136/qshc.7.3.149. 10185141 PMC2483604

[12] Improvement IfH , How to Improve: Model for Improvement, https://www.ihi.org/resources/how-improve-model-improvement.

[13] What is Lean Healthcare?, Catalyst Carryover. (2018) 4, no. 2.

[14] Glasgow R. E. , Vogt T. M. , and Boles S. M. , Evaluating the Public Health Impact of Health Promotion Interventions: the RE-AIM Framework, American Journal of Public Health. (1999) 89, no. 9, 1322–1327, 10.2105/ajph.89.9.1322. 10474547 PMC1508772

[15] Michie S. , Johnston M. , Abraham C. , Lawton R. , Parker D. , and Walker A. , Making Psychological Theory Useful for Implementing Evidence Based Practice: A Consensus Approach, Quality and Safety in Health Care. (2005) 14, no. 1, 26–33, 10.1136/qshc.2004.011155. 15692000 PMC1743963

[16] Atkins L. , Francis J. , Islam R. et al., A Guide to Using the Theoretical Domains Framework of Behaviour Change to Investigate Implementation Problems, Implementation Science. (2017) 12, no. 1, 10.1186/s13012-017-0605-9. PMC5480145 28637486

[17] Toronto U. H. , The Select Tool, 2019.

[18] Howlin C. and Rooney B. , The Cognitive Mechanisms in Music Listening Interventions for Pain: a Scoping Review, Journal of Music Therapy. (2020) 57, no. 2, 127–167, 10.1093/jmt/thaa003. 32249313

[19] Santos K. V. G. D. , Dantas J. K. D. S. , Fernandes T. E. D. L. et al., Music to Relieve Pain and Anxiety in Cardiac Catheterization: A Systematic Review and meta-analysis, Heliyon. (2024) 10, no. 13, 10.1016/j.heliyon.2024.e33815. PMC11263635 39044980

[20] Pelletier C. L. , The Effect of Music on Decreasing Arousal due to Stress: A Meta-Analysis, Journal of Music Therapy. (2004) 41, no. 3, 192–214, 10.1093/jmt/41.3.192. 15327345

[21] SEW , VB , and CP , Physiological Influences of Music in Perception and Action, 2022, Cambridge University Press.

[22] Nanchen D. , Resting Heart Rate: What is Normal?, Heart. (2018) 104, no. 13, 1048–1049, 10.1136/heartjnl-2017-312731. 29382691

[23] Ooishi Y. , Mukai H. , Watanabe K. , Kawato S. , and Kashino M. , Increase in Salivary Oxytocin and Decrease in Salivary Cortisol After Listening to Relaxing slow-tempo and Exciting fast-tempo Music, PLoS One. (2017) 12, no. 12. 10.1371/journal.pone.0189075 PMC5718605 29211795

[24] Kim S. , Gäbel C. , Aguilar-Raab C. , Hillecke T. , and Warth M. , Affective and Autonomic Response to Dynamic Rhythmic Entrainment – Mechanisms of a Specific Music Therapy Factor, The Arts in Psychotherapy. (2018) 60, 48–54, 10.1016/j.aip.2018.06.002.

[25] Vroom V. H. , Work and Motivation, 1964, Wiley.

[26] StataCorp , Stata Statistical Software: Release 19, 2025, StataCorp LLC.

[27] Johnson A. A. , Berry A. , Bradley M. et al., Examining the Effects of Music-Based Interventions on Pain and Anxiety in Hospitalized Children: An Integrative Review, Journal of Pediatric Nursing. (2021) 60, 71–76, 10.1016/j.pedn.2021.02.007. 33626485

[28] Lee H. Y. , Nam E. S. , Chai G. J. , and Kim D. M. , Benefits of Music Intervention on Anxiety, Pain, and Physiologic Response in Adults Undergoing Surgery: A Systematic Review and Meta-Analysis, Asian Nursing Research. (2023) 17, no. 3, 138–149, 10.1016/j.anr.2023.05.002. 37276961

[29] Strudwick G. , Jeffs L. , Kemp J. et al., Identifying and Adapting Interventions to Reduce Documentation Burden and Improve Nurses’ Efficiency in Using Electronic Health Record Systems (The IDEA Study): Protocol for a Mixed Methods Study, BMC Nursing. (2022) 21, no. 1, 10.1186/s12912-022-00989-w. PMC9351241 35927701

[30] Scala E. , Patterson B. J. , Stavarski D. H. , and Mackay P. , Engagement in Research: A Clinical Nurse Profile and Motivating Factors, Journal for Nurses in Professional Development. (2019) 35, no. 3, 137–143, 10.1097/nnd.0000000000000538. 30829742

[31] Alomari A. , Wilson V. , and Lewis J. , Action Research: A Mechanism for Active Engagement of Clinical Nurses in a Program of Research, Nursing and Health Sciences. (2020) 22, no. 3, 539–547, 10.1111/nhs.12690. 32107834

[32] Mondanaro J. F. , Homel P. , Lonner B. , Shepp J. , Lichtensztein M. , and Loewy J. V. , Music Therapy Increases Comfort and Reduces Pain in Patients Recovering From Spine Surgery, American Journal of Orthopedics. (2017) 46, no. 1, E13–e22. 28235116

[33] Kühlmann A. Y. , De Rooij A. , Kroese L. F. , Van Dijk M. , Hunink M. G. , and Jeekel J. , Meta-Analysis Evaluating Music Interventions for Anxiety and Pain in Surgery, Journal of British Surgery. (2018) 105, no. 7, 773–783, 10.1002/bjs.10853. PMC6175460 29665028

[34] Santiváñez-Acosta R. , Tapia-López E. D. , and Santero M. , Music Therapy in Pain and Anxiety Management During Labor: A Systematic Review and Meta-Analysis, Medicina. (2020) 56, no. 10, 10.3390/medicina56100526. PMC7599829 33050409

