# Supplementary material for: Nurse Implementation of Music for Pain Relief in the Inpatient Setting
Source: Nurs Res Pract. 2026 Aug 29;2026:7578595. doi: 10.1155/nrp/7578595 (PMC13525375; doi:10.1155/nrp/7578595)
Supplement: Supplementary file 1 [file NRP-2026-7578595-s001.docx]

Supplemental Appendix A: Examples of Curated Playlists for the Music for Pain Relief Research Study

- Relaxation Music for Children (34 min) - <https://open.spotify.com/playlist/6F3NDdNatPrRD8ujzHeMkI?si=TBEbNhW2Tmmr5qnhWfkTLQ>

- Acoustic Guitar (31 min) - <https://open.spotify.com/playlist/1CbZ7GTCNCY4FevCq1aWVk?si=FRh6njiwRreGFoEkiH1KWg>

- Acoustic Guitar + Ocean Sounds (35 min) - <https://open.spotify.com/playlist/7h7poXg9SnTjOHOCyrfSDe?si=DgDNb1VKS4KN1P6eAMjCAw>

- Acoustic Guitar + Cello (31 min) - <https://open.spotify.com/playlist/5WPi4p4ZBOP5GpalAGtKkT?si=MwM1UMqgSyiFQfokemByKg>
