# Supplementary material for: Nurse Implementation of Music for Pain Relief in the Inpatient Setting
Source: Nurs Res Pract. 2026 Aug 29;2026:7578595. doi: 10.1155/nrp/7578595 (PMC13525375; doi:10.1155/nrp/7578595)
Supplement: Supplementary file 2 [file NRP-2026-7578595-s002.docx]

Supplemental Figure 1. Stratified documented monthly use of music interventions by intervention phase

No. of times music intervention was used

|  | Adult | Child |
| --- | --- | --- |
| Feb 2021 | 11 | 1 |
| Mar 2021 | 6 | 1 |
| Apr 2021 | 13 | 1 |
| May 2021 | 1 | 2 |
| Jul 2021 | 9 | 2 |
| Aug 2021 | 10 | 0 |
| Sept 2021 | 19 | 0 |
| Oct 2021 | 17 | 5 |
| Nov 2021 | 13 | 4 |
| Dec 2021 | 13 | 4 |
| Jan 2022 | 20 | 0 |
| Feb 2022 | 13 | 6 |
| Mar 2022 | 11 | 6 |
| Apr 2022 | 29 | 2 |
