# Supplementary material for: Nurse Implementation of Music for Pain Relief in the Inpatient Setting
Source: Nurs Res Pract. 2026 Aug 29;2026:7578595. doi: 10.1155/nrp/7578595 (PMC13525375; doi:10.1155/nrp/7578595)
Supplement: Supplementary file 3 [file NRP-2026-7578595-s003.docx]

Supplemental Table 1. Stratified pain scores before and after music intervention

|  |  | Within 1 hour before music intervention | |  | Within 3 hours after music intervention | |  |
| --- | --- | --- | --- | --- | --- | --- | --- |
|  | n | Mean | SD |  | Mean | SD | P value* |
| All patients | | | | | | | |
| All locations | 43 | 5.14 | 2.99 |  | 5.33 | 3.50 | 0.868 |
| Abdomen | 7 | 6.57 | 1.99 |  | 7.43 | 2.94 | 0.610 |
| Extremities | 6 | 3.83 | 1.83 |  | 4.67 | 2.94 | 0.831 |
| Generalized | 4 | 8.00 | 2.83 |  | 7.50 | 3.79 | 0.317 |
| HEENT | 7 | 4.79 | 1.63 |  | 5.57 | 3.69 | 0.553 |
| Thorax | 6 | 5.50 | 2.59 |  | 6.17 | 3.19 | 0.898 |
| Unknown | 13 | 4.12 | 3.96 |  | 3.31 | 3.35 | 0.246 |
| Adults (aged 18+ years) | | | | | | | |
| All locations | 35 | 5.19 | 3.03 |  | 5.40 | 3.67 | 0.967 |
| Abdomen | 5 | 5.60 | 1.14 |  | 7.00 | 3.46 | 0.416 |
| Extremities | 4 | 4.50 | 1.91 |  | 6.00 | 2.71 | 0.462 |
| Generalized | 4 | 8.00 | 2.83 |  | 7.50 | 3.79 | 0.317 |
| HEENT | 5 | 4.60 | 1.52 |  | 5.20 | 4.44 | 0.686 |
| Thorax | 4 | 6.75 | 2.22 |  | 7.75 | 2.63 | 0.842 |
| Unknown | 13 | 4.12 | 3.96 |  | 3.31 | 3.35 | 0.246 |
| Children (aged < 18 years) | | | | | | | |
| All locations | 8 | 4.94 | 2.96 |  | 5.00 | 2.83 | 0.771 |
| Abdomen | 2 | 9.00 | 1.41 |  | 8.50 | 0.71 | 0.655 |
| Extremities | 2 | 2.50 | 0.71 |  | 2.00 | 0.00 | 0.317 |
| Generalized | 0 |  |  |  |  |  |  |
| HEENT | 2 | 5.25 | 2.47 |  | 6.50 | 0.71 | 0.655 |
| Thorax | 2 | 3.00 | 0.00 |  | 3.00 | 0.00 | – |
| Unknown | 0 |  |  |  |  |  |  |
| *From Wilcoxon signed rank tests | | |  |  |  |  |  |
